# Errata

**Published:** 1895-04

**Authors:** 


[*Errata.*—Page 298 (December number of JOURNAL), line seventeen from top, for *Zehender*, *of Munich*, read *Hansen-grut*, *of Copenhagen ;* page 485 (March number), line three from top, for *inferior*, read *superior ;* line nine from top, for *180*, read *180°* ; line six from bottom, for *2°*, read *2.00 D.* ; page 487, line three from top, for *Doyer*, read *Doijer.*—A. A. H.]

